# Supplementary material for: A common class of transcripts with 5′-intron depletion, distinct early coding sequence features, and N1-methyladenosine modification
Source: RNA. 2017 Mar;23(3):270–83. doi: 10.1261/rna.059105.116 (PMC5311483; doi:10.1261/rna.059105.116)
Supplement: Supplemental Material [file supp_23_3_270__index.html]

Supplemental Material 

# A common class of transcripts with 5′-intron depletion, distinct early coding sequence features, and *N*1-methyladenosine modification

## Supplemental Material

**Files in this Data Supplement:**

- Supplemental Figure S1.pdf
- Supplemental Figure S2.pdf
- Supplemental Figure S3.pdf
- Supplemental Figure S4.pdf
- Supplemental Figure S5.pdf
- Supplemental Figure S6.pdf
- Supplemental Figure S7.pdf
- Supplemental Figure S8.pdf
- Supplemental Figure S9.pdf
- Supplemental\_Legends.docx
- Table\_S1.txt
- Table\_S2.tsv
- Table\_S3.docx
- Table\_S4.csv
