# Supplementary figures and images for: A common class of transcripts with 5′-intron depletion, distinct early coding sequence features, and N1-methyladenosine modification
Source: RNA. 2017 Mar;23(3):270–83. doi: 10.1261/rna.059105.116 (PMC5311483; doi:10.1261/rna.059105.116)

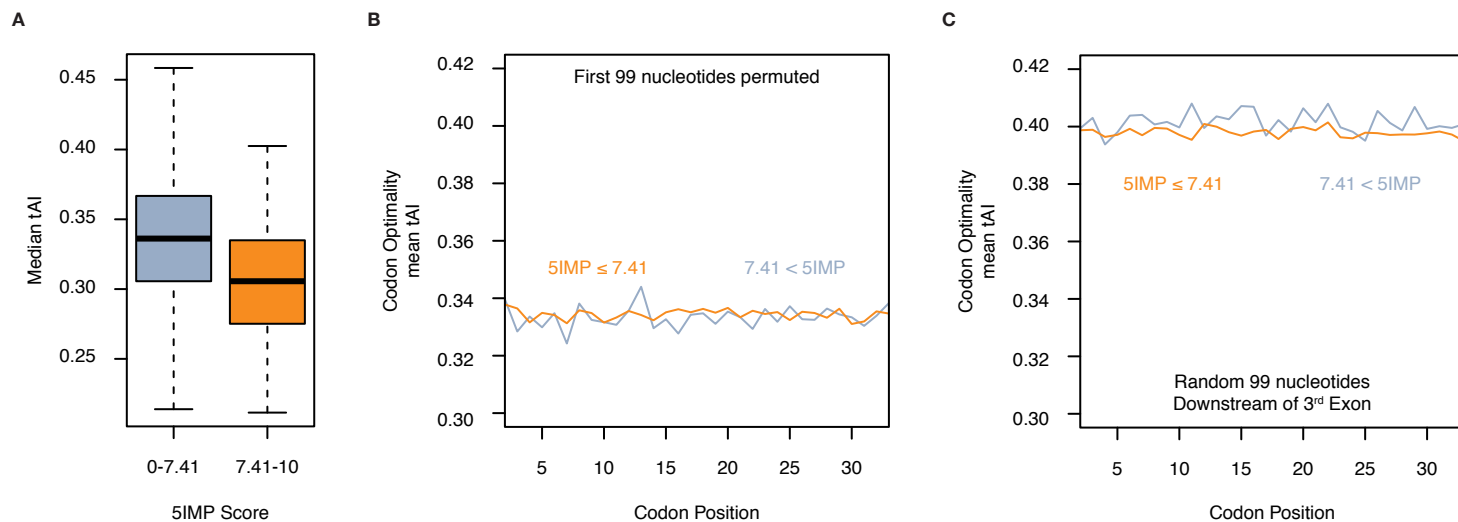

Supplement: Supplemental Material [file supp_059105.116_Figure_S1.pdf]

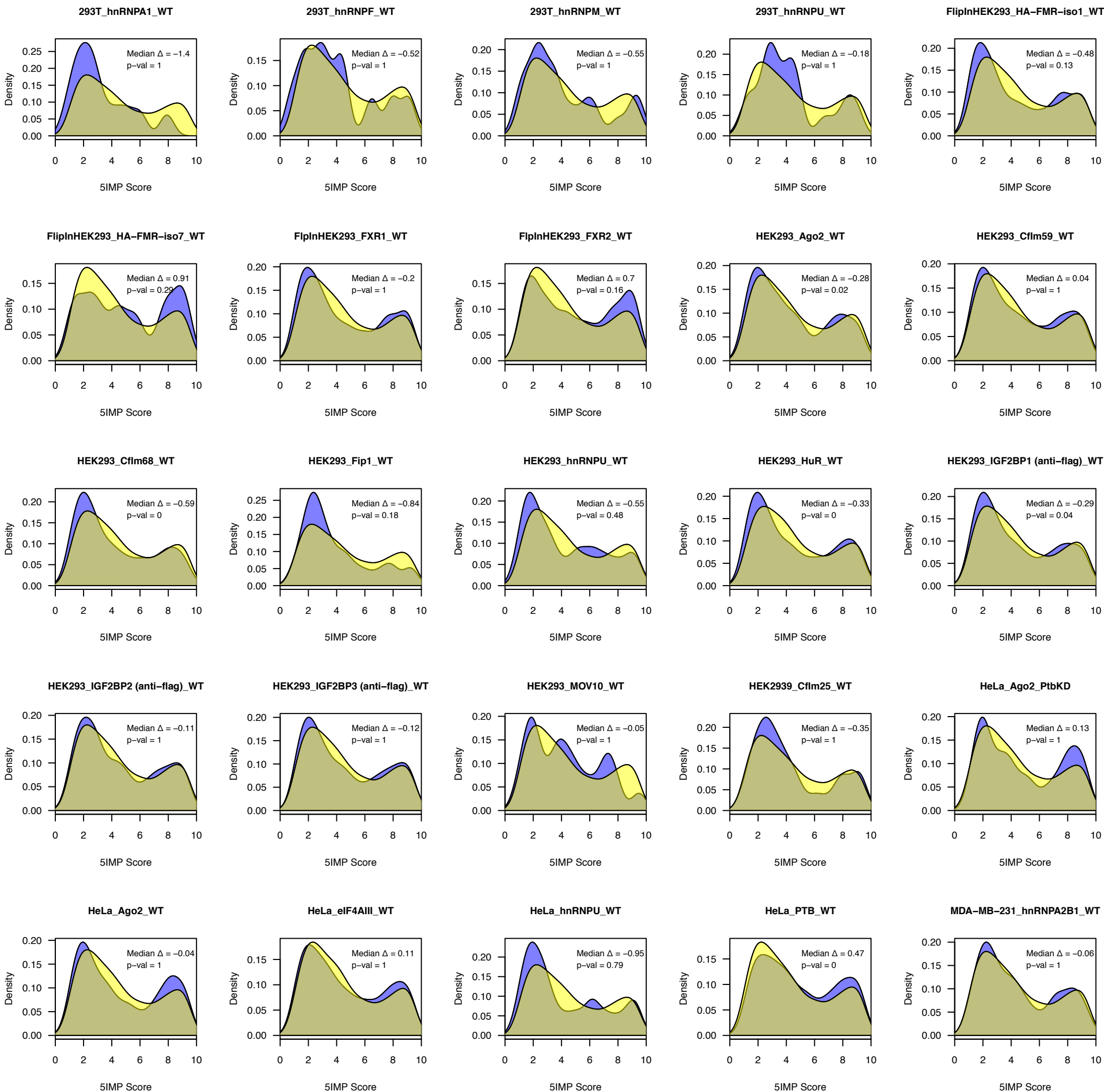

Supplement: Supplemental Material [file supp_059105.116_Figure_S2.pdf]

A

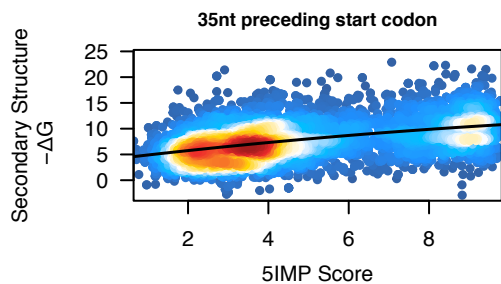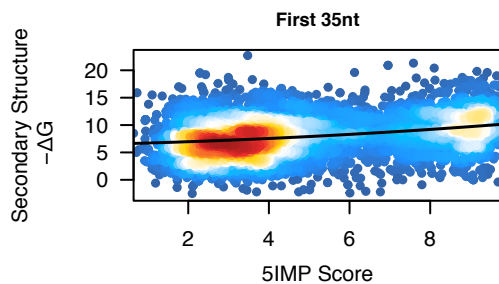

B

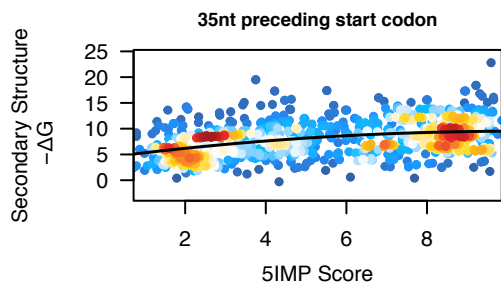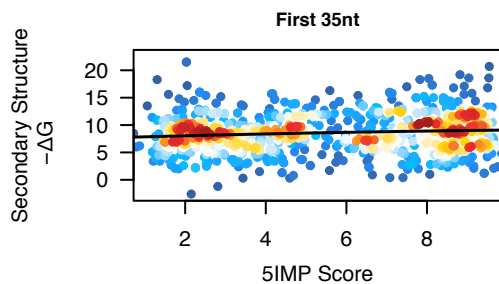

C

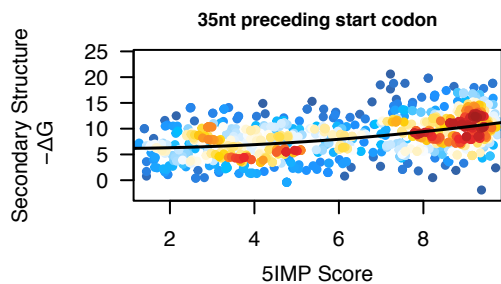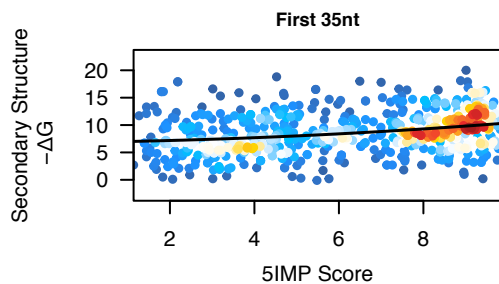

D

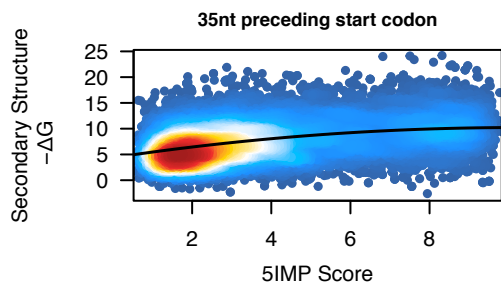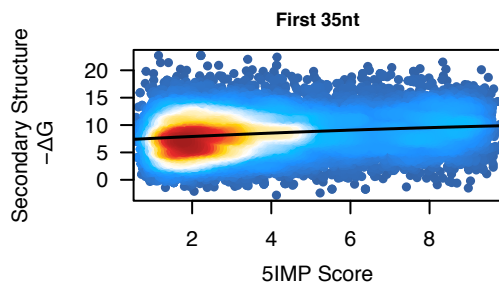

Supplement: Supplemental Material [file supp_059105.116_Figure_S3.pdf]

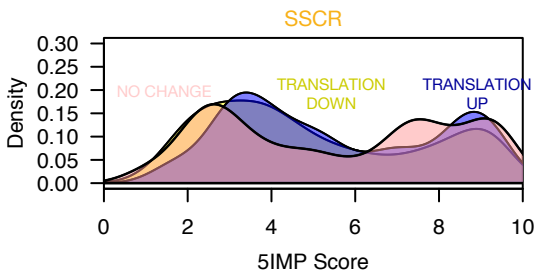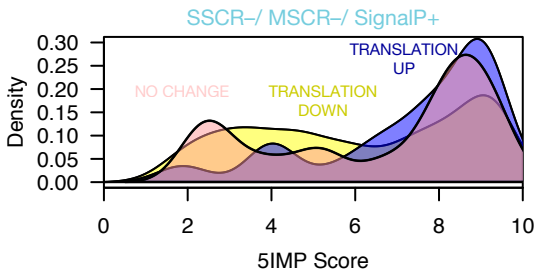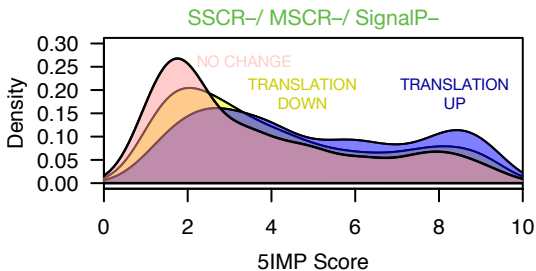

Supplement: Supplemental Material [file supp_059105.116_Figure_S4.pdf]

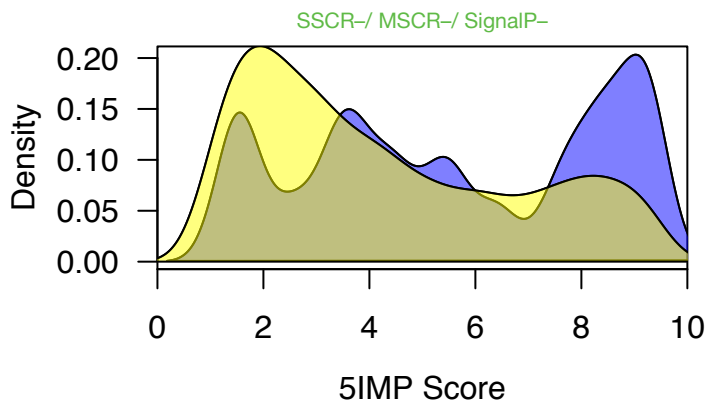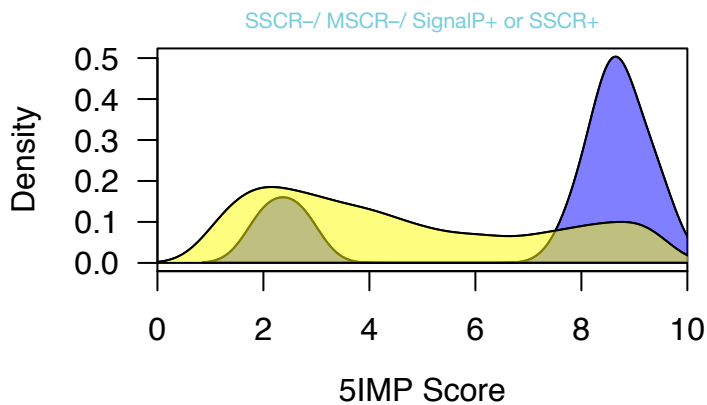

Supplement: Supplemental Material [file supp_059105.116_Figure_S5.pdf]

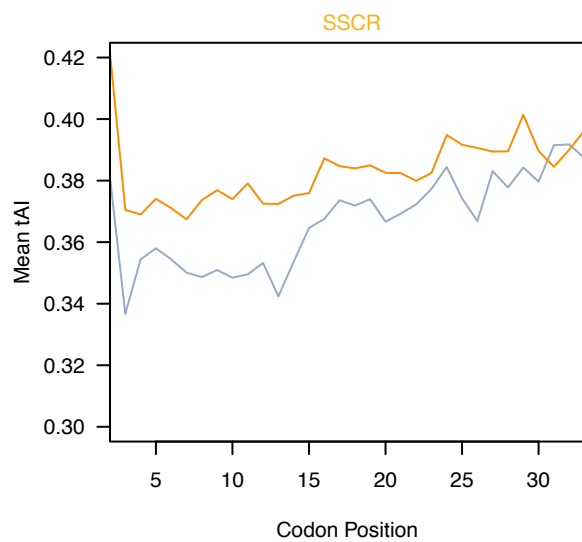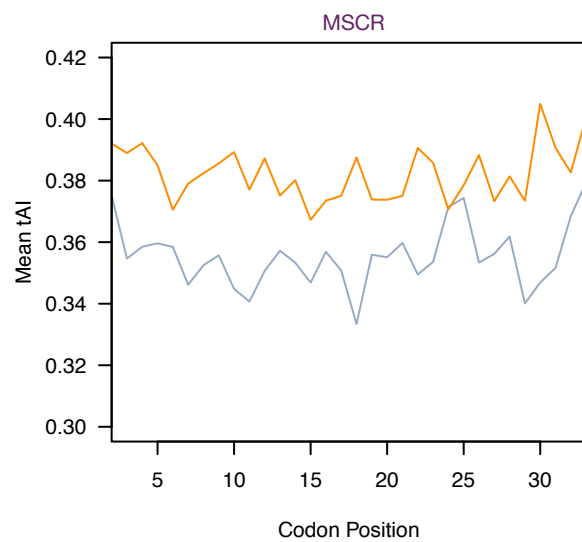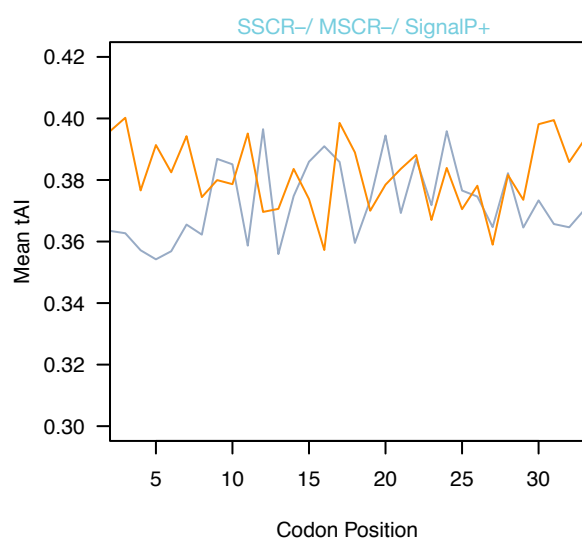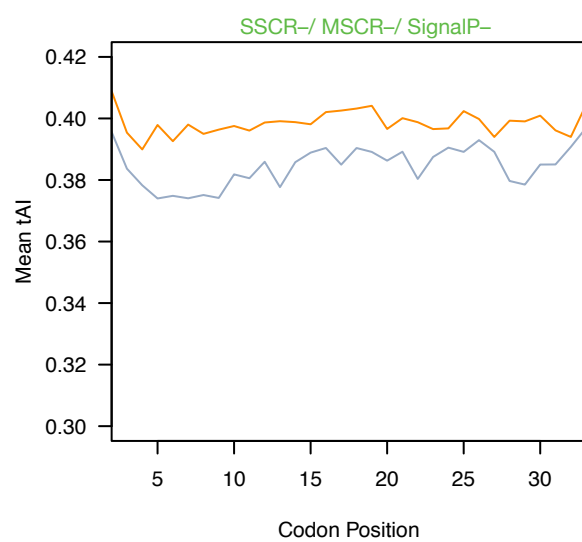

Supplement: Supplemental Material [file supp_059105.116_Figure_S6.pdf]

SSCR

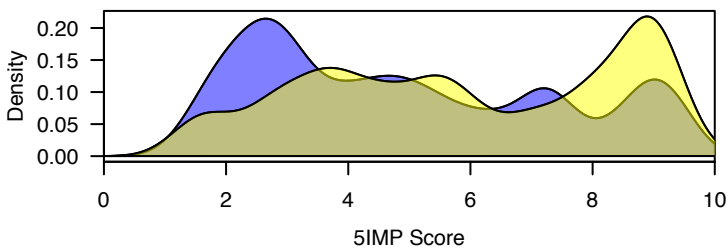

MSCR

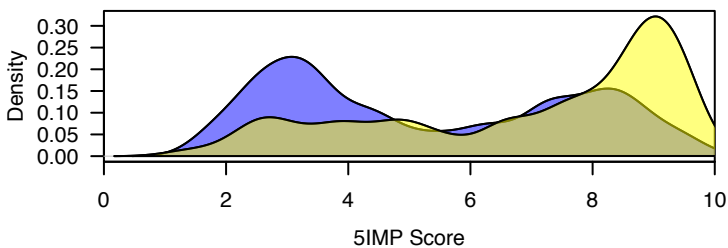

SSCR-/ MSCR-/ SignalP+

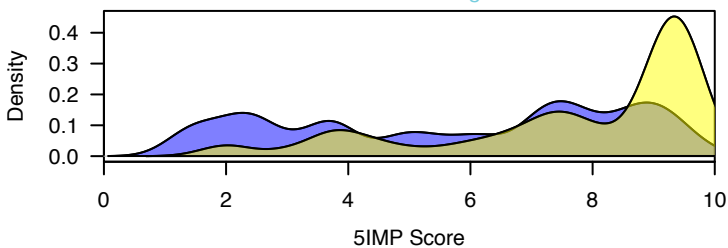

SSCR-/ MSCR-/ SignalP-

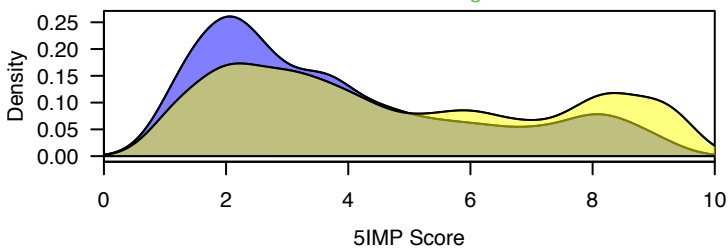

Supplement: Supplemental Material [file supp_059105.116_Figure_S7.pdf]

SSCR

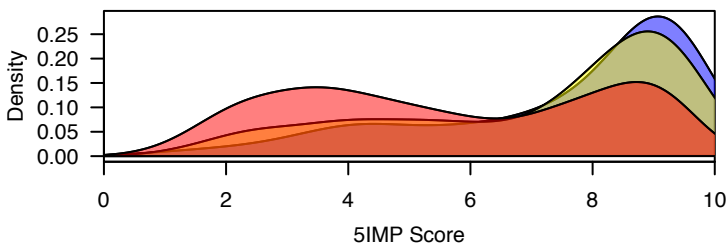

MSCR

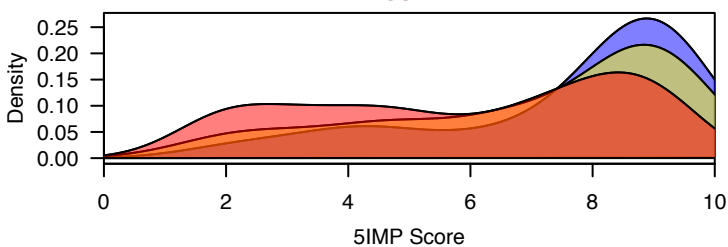

SSCR- / MSCR- / SignalP+

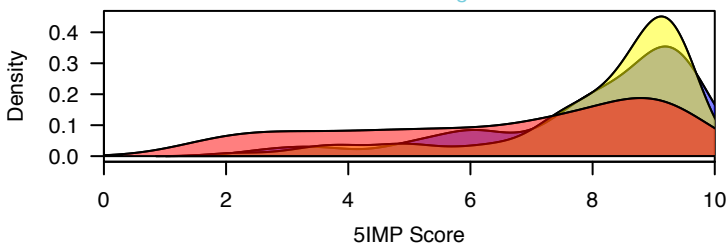

SSCR- / MSCR- / SignalP-

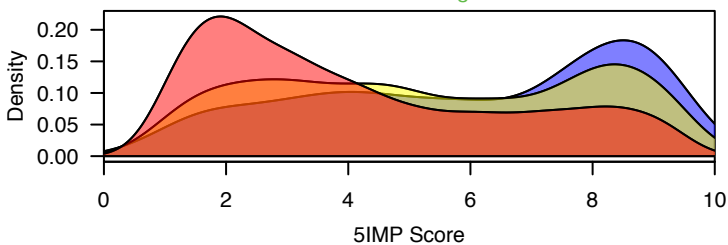

Supplement: Supplemental Material [file supp_059105.116_Figure_S8.pdf]

SSCR

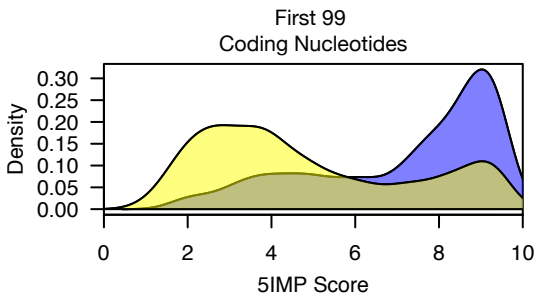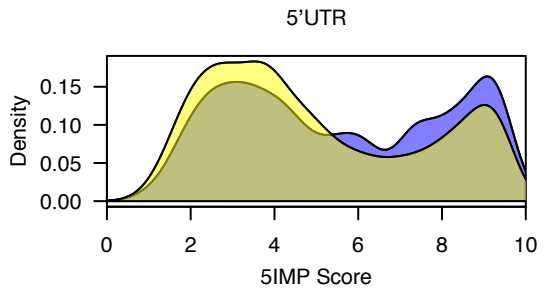

MSCR

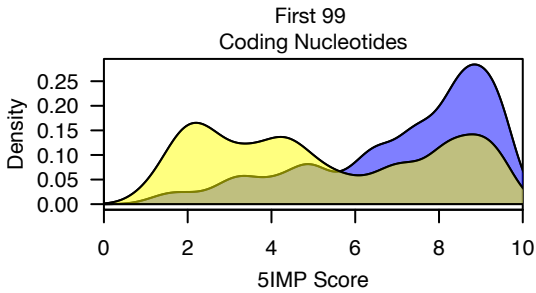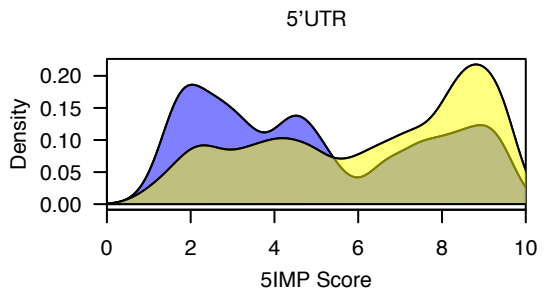

SSCR-/ MSCR-/ SignalP+

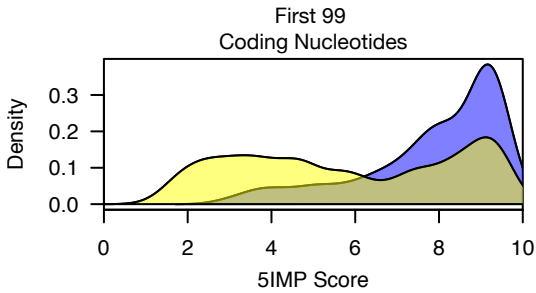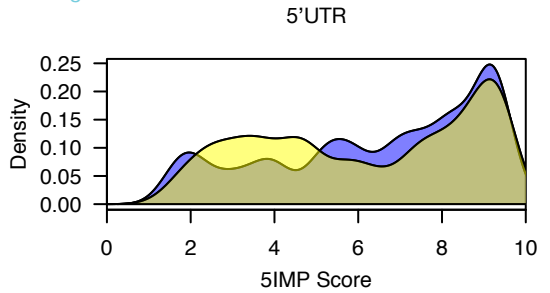

SSCR-/ MSCR-/ SignalP-

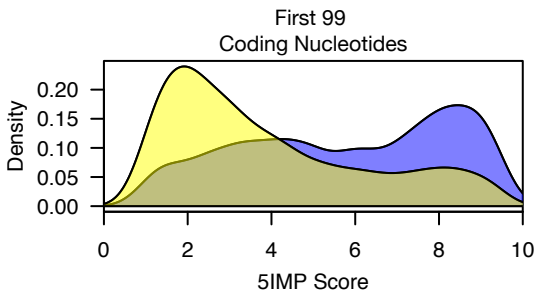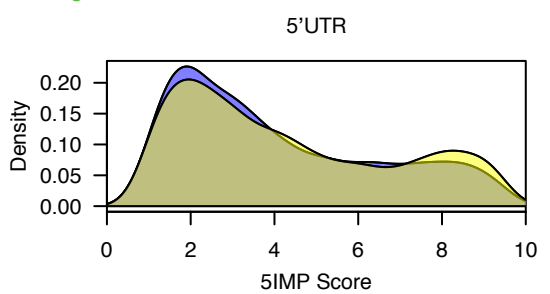

Supplement: Supplemental Material [file supp_059105.116_Figure_S9.pdf]
