# Supplementary material for: A common class of transcripts with 5′-intron depletion, distinct early coding sequence features, and N1-methyladenosine modification
Source: RNA. 2017 Mar;23(3):270–83. doi: 10.1261/rna.059105.116 (PMC5311483; doi:10.1261/rna.059105.116)
Supplement: Supplemental Material [file supp_059105.116_Supplemental_Legends.docx]

**Supporting Information**

**Figure S1 | Association between 5IMP scores and codon optimality is restricted to the first 30 amino acids and is not explained by nucleotide content.** **(a)** 5IM transcripts tend to have less optimal codons in their first 30 amino acids as measured by tRNA adaptation index (tAI). The median tAI for each transcript was calculated and transcripts were grouped by their 5IMP scores. The distribution of median tAI was plotted as a boxplot. **(b)** We permuted the nucleotides of the first 99 nucleotides and found that the relationship between 5IMP and tAI was lost. This result suggested that nucleotide composition alone doesn’t explain the relationship between 5IMP and tAI. **(c)** We used the previously described negative control sequences, each derived from a single randomly chosen in-frame ‘window’ downstream of the 3rd exon from one of the evaluated transcripts. We found no relationship between 5IMP score and tAI in these regions suggesting that the observed association is restricted to the early coding region.

**Figure S2 | Targets of various RNA-binding proteins are not enriched in high 5IMP scores (a)** Targets of various RNA-binding proteins were identified using a uniform analysis pipeline (Methods). The 5IMP scores for targets of each RBP were compared to non-target transcripts using Wilcoxon Rank Sum test. None of the tested RBP target sets had a Bonferroni adjusted p-value < 0.05 and a median 5IMP score difference > 1 when compared to non-targets. Each figure panel is labeled with cell type, targeted RBP, treatment (WT = no treatment or PtbKD = shRNA knock-down of PTB).

**Figure S3 | The 5IM classifier score was positively correlated with the propensity for mRNA structure near 5’ cap and the start codon** **(a)** For each transcript, 35 nucleotides immediately upstream of the start codon and 35 nucleotides following the 5’cap were used to calculate –ΔG. 5IMP score was plotted against –ΔG for SSCR transcripts. **(b)** 5IMP score was plotted against –ΔG for MSCR transcripts. **(c)** 5IMP score was plotted against –ΔG for SignalP^+^ transcripts. **(b)** 5IMP score was plotted against –ΔG for S^–^/M^–^/SignalP^–^ transcripts.

**Figure S4 | Translationally upregulated transcripts in response to eIF4E overexpression are enriched for higher 5IMP scores.** **(a)** Analysis shown in Figure 3C was repeated for different subclasses of transcripts. There were no translationally down regulated transcripts among the MSCR class.

**Figure S5 | Association between 5IMP scores and transcripts with non-AUG start codons**. Transcripts with non-AUG start codons (blue) exhibited significantly higher 5IMP scores than transcripts with a canonical ATG start codon (yellow). There were no MSCR transcripts with annotated non-AUG start codons. Among SignalP^+^ transcripts, there was only one with a non-AUG start codon and was merged with SSCR transcripts.

**Figure S6 | Higher 5IMP scores are associated with lower codon optimality among S^–^/M^–^/SignalP^–^ transcripts.** For all transcripts within each 5IMP score category (blue-high, orange-low), the mean tAI was calculated at each codon position (2-33). Start codon was not shown.

**Figure S7 | Association between 5IMP scores and the number of ribosomes per mRNA does not depend on transcript class.** Transcripts with translation efficiency one standard deviation below the mean (“LOW” translation, yellow) and one standard deviation higher than the mean (“HIGH” translation, blue) were identified using ribosome profiling and RNA-Seq data from human lymphoblastoid cell lines (Methods).

**Figure S8 | Association between 5IMP scores and non-canonical Exon Junction Complex (EJC) binding sites does not depend on transcript class.** 5IMP score distribution for transcripts with zero (red), one (yellow), two or more (blue) non-canonical EJC binding sites in the first 99 coding nucleotides reveals that transcripts with high 5IMP scores frequently harbor non-canonical EJC binding sites regardless of transcript class.

**Figure S9 | Association between 5IMP scores and mRNAs with early coding region m^1^A modifications does not depend on transcript class.** Transcripts with m^1^A modifications (blue) in the first 99 coding nucleotides (left) exhibit significant enrichment for 5IM transcripts and have higher 5IMP scores than transcripts without m^1^A modifications in the first 99 coding nucleotides (yellow). This enrichment is absent when transcripts with m^1^A modifications (blue) in the 5’UTR (right) are considered.

**Table S1- |** List of features used by the 5IM classifier.

**Table S2- |** 5IMP scores of all human transcripts.

**Table S3- |** List of functional features tested for association with 5IMP scores.

**Table S4- |** GEO accession numbers of datasets used for determining RNA-binding protein targets.
