# Supplementary material for: A common class of transcripts with 5′-intron depletion, distinct early coding sequence features, and N1-methyladenosine modification
Source: RNA. 2017 Mar;23(3):270–83. doi: 10.1261/rna.059105.116 (PMC5311483; doi:10.1261/rna.059105.116)
Supplement: Supplemental Material [file supp_059105.116_Table_S1.txt]

Table S1. List of Features Used in Training the Random Forest

| FEATURE CLASS | PARTICULAR FEATURES USED |
| --- | --- |
| Ratio of Amino Acids | Number of Leucines / Number of Isoleucines |
|  | Number of Arginines / Number of Lysines |
|  | Number of Aspartates / Number of Glutamates |
|  | Number of Phenylalanines / Number of Tyrosines |
|  | Number of Leucines / Number of Valines |
|  | Number of Valines / Number of Isoleucines |
|  | Number of Glutamines / Number of Glutamates |
| Nucleotide Content | Percent Adenines |
|  | Percent Thymines |
|  | Length of the Longest Track Lacking Any Adenines |
| Codon Preferences | Ratio of Non-Adenine Codons to Adenine Codons |
|  | Ratio of Non-Thymine Codons to Thymine Codons |
| Motif Score and Position | MoAn – 12 features including PSSM scores and positions of the top two occurrences for each of 3 motifs discovered by MoAn [[44]](https://paperpile.com/c/Xqhywn/s67k) |
|  | DEME – 4 features including PSSM scores and positions of the top two occurrences for 1 motif discovered by DEME[[43]](https://paperpile.com/c/Xqhywn/YbiX) |
|  | AlignACE – 8 features including PSSM scores and positions of the top two occurrences for each of 2 motifs discovered by AlignACE [[42]](https://paperpile.com/c/Xqhywn/EZod) |
