# Supplementary material for: A common class of transcripts with 5′-intron depletion, distinct early coding sequence features, and N1-methyladenosine modification
Source: RNA. 2017 Mar;23(3):270–83. doi: 10.1261/rna.059105.116 (PMC5311483; doi:10.1261/rna.059105.116)
Supplement: Supplemental Material [file supp_059105.116_Table_S3.docx]

**Table S3. List of features tested for association with 5IMP scores**

RNA substrates of Adenosine to Inosine Editing

Transcripts that contain AU-rich elements

Transcripts that are localized to dendrites

Transcript that exhibit differential expression across conditions (retrieved from ArrayExpress)

Transcripts encoding housekeeping proteins

Transcripts annotated to contain IRES sequences

Transcripts that are localized to post-synaptic membrane

Transcripts annotated to contain non-AUG start codons

Transcripts containing terminal oligo-pyrimidine track

Transcripts regulated by mTOR

Transcripts with intronic TDP-43 binding sites

Transcripts with exonic TDP-43 binding sites

Transcripts with both intronic and exonic TDP-43 binding sites

Transcripts upregulated upon UAP56 knockdown

Transcripts downregulated upon UAP56 knockdown

Transcripts upregulated upon URH49 knockdown

Transcripts downregulated upon URH49 knockdown

Transcripts translationally upregulated upon eIF4E overexpression

Transcripts translationally downregulated upon eIF4E overexpression

Transcripts transcriptionally upregulated upon eIF4E overexpression

Transcripts transcriptionally downregulated upon eIF4E overexpression

Transcripts associated with Staufen-1

Transcripts associated with Staufen-2

Human orthologs of yeast transcripts localized to the mitochondria

Transcripts containing N^1^-methyladenosines

Transcripts annotated with Gene Ontology terms or KEGG pathways

Transcripts that are enriched in the Brij-sensitive ER fraction

Transcripts that are enriched in the Brij-resistant ER fraction

Transcripts that are bound by RNA-binding proteins as measured by CLIP-seq variants (hnRNPA1, hnRNPF, hnRNPM, hnRNPU, Ago2, hnRNPU, HuR, IGF2BP1, IGF2BP2, IGF2BP3, FMR1, FXR1, FX2, eIF4AIII, PTB, IGF2BP1, Ago3, Ago4, MOV10, Fip1, CF Im68, CF Im59, CF Im25, and hnRNPA2B1)

Transcripts that are bound by the Exon Junction Complex

5’ UTR length

Codon optimality as measured by tRNA Adaptation Index (tAI)

Decay rates of mRNAs

Secondary structure near the 5’cap

Secondary structure near the start codon

Splice site strength of the 5’UTR intron

Ribosomes per mRNA molecule as measured by ribosome Profiling

Protein and mRNA levels in 4 cell lines

Half-life of proteins as measured by “bleach-chase”
